# The way to AI-controlled synthesis: how far do we need to go?

**DOI:** 10.1039/d2sc04419f

**Published:** 2022-09-27

**Authors:** Wei Wang, Yingwei Liu, Zheng Wang, Gefei Hao, Baoan Song

**Affiliations:** State Key Laboratory Breeding Base of Green Pesticide and Agricultural Bioengineering, Key Laboratory of Green Pesticide and Agricultural Bioengineering, Ministry of Education, Research and Development Center for Fine Chemicals, Guizhou University Guiyang 550025 P. R. China gefei_hao@foxmail.com; State Key Laboratory of Public Big Data, Guizhou University Guiyang 550025 P. R. China

## Abstract

Chemical synthesis always plays an irreplaceable role in chemical, materials, and pharmacological fields. Meanwhile, artificial intelligence (AI) is causing a rapid technological revolution in many fields by replacing manual chemical synthesis and has exhibited a much more economical and time-efficient manner. However, the rate-determining step of AI-controlled synthesis systems is rarely mentioned, which makes it difficult to apply them in general laboratories. Here, the history of developing AI-aided synthesis has been overviewed and summarized. We propose that the hardware of AI-controlled synthesis systems should be more adaptive to execute reactions with different phase reagents and under different reaction conditions, and the software of AI-controlled synthesis systems should have richer kinds of reaction prediction modules. An updated system will better address more different kinds of syntheses. Our viewpoint could help scientists advance the revolution that combines AI and synthesis to achieve more progress in complicated systems.

## Abstract 2

It is still a long march for AI-controlled synthesis to enter into general laboratories. Flaws in the architecture of AI-controlled synthesis systems must be overcome.

## Graphical abstract

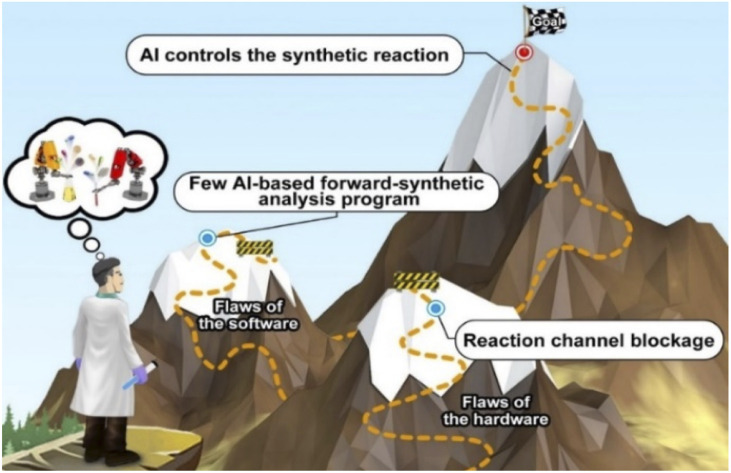

## 1 Introduction

After decades of pioneering research in academia, it has been shown that chemical synthesis has long been an integral part of human life.^1^ Synthesis is everywhere; everything that can be heard, seen, smelled, tasted, and touched involves synthesis. However, completing the chemical synthesis process faster, safer, more economically and more efficiently is still an issue of concern all over the world. Nonetheless, traditional research methods may not be sufficiently efficient.^2,3^ Thus, people propose using AI techniques to assist with chemical synthesis.

**Fig. 1:**
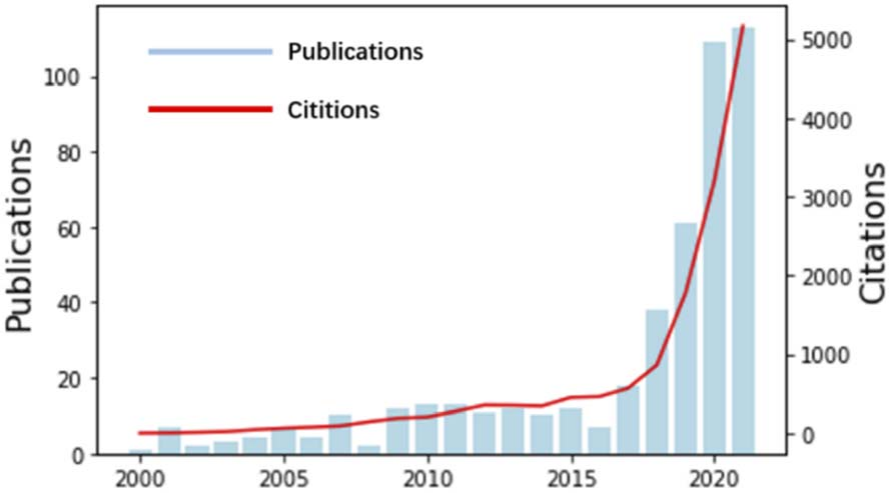
Research on AI-aided chemical synthesis is increasingly popular.

AI is based on computational machines, and the theory and application of computational machines have a long history and have been applied gradually in many fields since the last century. In 1948, Claude Shannon reported that information can be encoded by binary systems, which launched the field of information theory and set the stage for the merger of data science and chemical synthesis.^4^ With years of developing electronic techniques, more AI algorithms have been developed; therefore, the applications are not restricted to the development of simple tools.^5–9^ As shown in Fig. 1, from 2000 to 2021, studies combining AI and chemical synthesis were increasingly carried out. Especially in this five-year period, regardless of publications or citations, they are increasing exponentially. Currently, AI applications can be realized by different methods (*e.g.*, support vector machines (SVMs),^10^ random forests (RFs),^11^ artificial neural networks (ANNs),^12^ convolutional neural networks (CNNs),^13^ recurrent neural networks (RNNs),^14^ and graph neural networks (GNNs)^15^). As shown in Table 1, AI algorithms are causing a rapid technological revolution in many fields of science, especially in the chemical field, with high process speed and high accuracy.^16–20^

**Table 1:** Some cases with different machine learning or AI methods

Methods	Classification	Case	Main work of case	Ref.
SVM	Kernel learning	Granda *et al.*	The reactivity of about 1000 combinations was predicted and the accuracy was more than 80%	16
RF	Decision tree	Ahneman *et al.*	The performance of C–N cross-coupling reactions was predicted by a RF module	17
CNN	ANN	Staker *et al.*	A structure prediction model was based on SMILES strings with more than 80% accuracy	18
RNN	ANN	Schwaller *et al.*	An attention-based model which is like the learning process of the human brain determined a better connection style to solve problems	19
GNN	ANN	Coley *et al.*	With the information on solvents, reactants and reagents, the model could fast predict the products of organic reactions in 100 ms	20

**Fig. 2:**
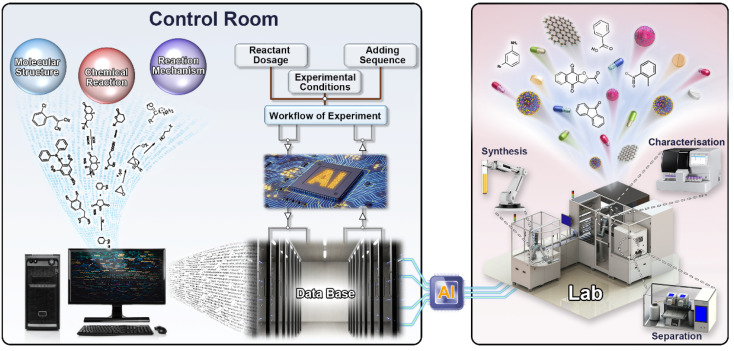
A mature AI-controlled chemical automatic synthesis laboratory (the software in the control room; the hardware in the lab; automation control components connecting them).

It is not a new concept that AI is used as a chemical assist-tool. Corey *et al.* employed AI techniques to generate chemical pathways for complex molecules in 1969.^21^ AI as an assist-tool is also a promising field of research with the potential to have a tremendous impact on drug discovery, industrial chemistry, and materials science.^22–25^ For example, Li *et al.* used a unified and fully automated process to complete the synthesis of 14 different small molecules.^26^ Mijalis *et al.* reported an automated method for synthesizing polypeptides. The amide bond formation time was only seven seconds, and the total synthesis of each amino acid residue only required 40 seconds.^27^ Coley *et al.* combined AI and a series of hardware, making synthesis automation take a leap forward. They predicted and automated the synthesis of 15 drugs or drug-like molecules in 96 hours with an AI-based model.^28^ Clayton *et al.* reported a multistep reaction automatic optimization platform that could provide substantial savings in time and resources. This AI-based method had the ability to simultaneously optimize multistep processes for multiple goals.^29^ Gómez-Bombarelli *et al.* used a computational method to guide all stages of a materials synthesis workflow. They successfully synthesized efficient molecular organic light-emitting diodes.^30^ Minato *et al.* utilized a robotic workflow modular wheel platform (MWP) to generate new heteromultinuclear metal clusters with high energy barriers for magnetization reversal. With the assistance of the MWP, the time cycle of discovering new clusters is shortened.^31^ Chemical synthesis combined with AI could produce revolutionary achievements. Moreover, AI has also been widely applied in the chemical synthesis field due to the following advantages: (1) increasing the efficiency of chemical synthesis; (2) saving time and manpower; (3) avoiding errors from manual operation; and (4) increasing personal security. Here, the concept of “AI-controlled synthesis” is proposed. An AI-controlled synthesis system is dependent on multiple AI-aided synthesis platforms; that is, the technique of AI-controlled synthesis is a combination of several techniques of AI-aided synthesis. Therefore, a mature AI-controlled synthesis system should be made from at least two parts (Fig. 2): software as a controller in the control room and hardware as an executor in the laboratory. However, AI-aided synthesis systems are only applicable in some specific laboratories that have developed their own AI-aided synthesis systems.^32–34^ Many general laboratories still use manual operations to complete synthesis. Thus, we are facing an issue. How long do we need to go to extend the applications of AI-controlled synthesis into general laboratories?

In this work, we overviewed the current workflow of AI-aided synthesis and summarized current applications. We found the key restrictive processes for AI-controlled synthesis in general laboratories, and we also proposed some perspectives to overcome these limiting factors. We believe that this work could help overcome the difficulties that hinder AI-controlled synthesis in general laboratories.

## 2 Hardware: executor of AI-controlled synthesis

The hardware of the AI-controlled synthesis system is the equivalent of chemists' hands during manual operation. It plays the role of an executor and liberates chemists' hands. Its configuration can be as simple as a machine arm or be quite complex as a large automation platform.

### 2.1 The configuration of the hardware

**Fig. 3:**
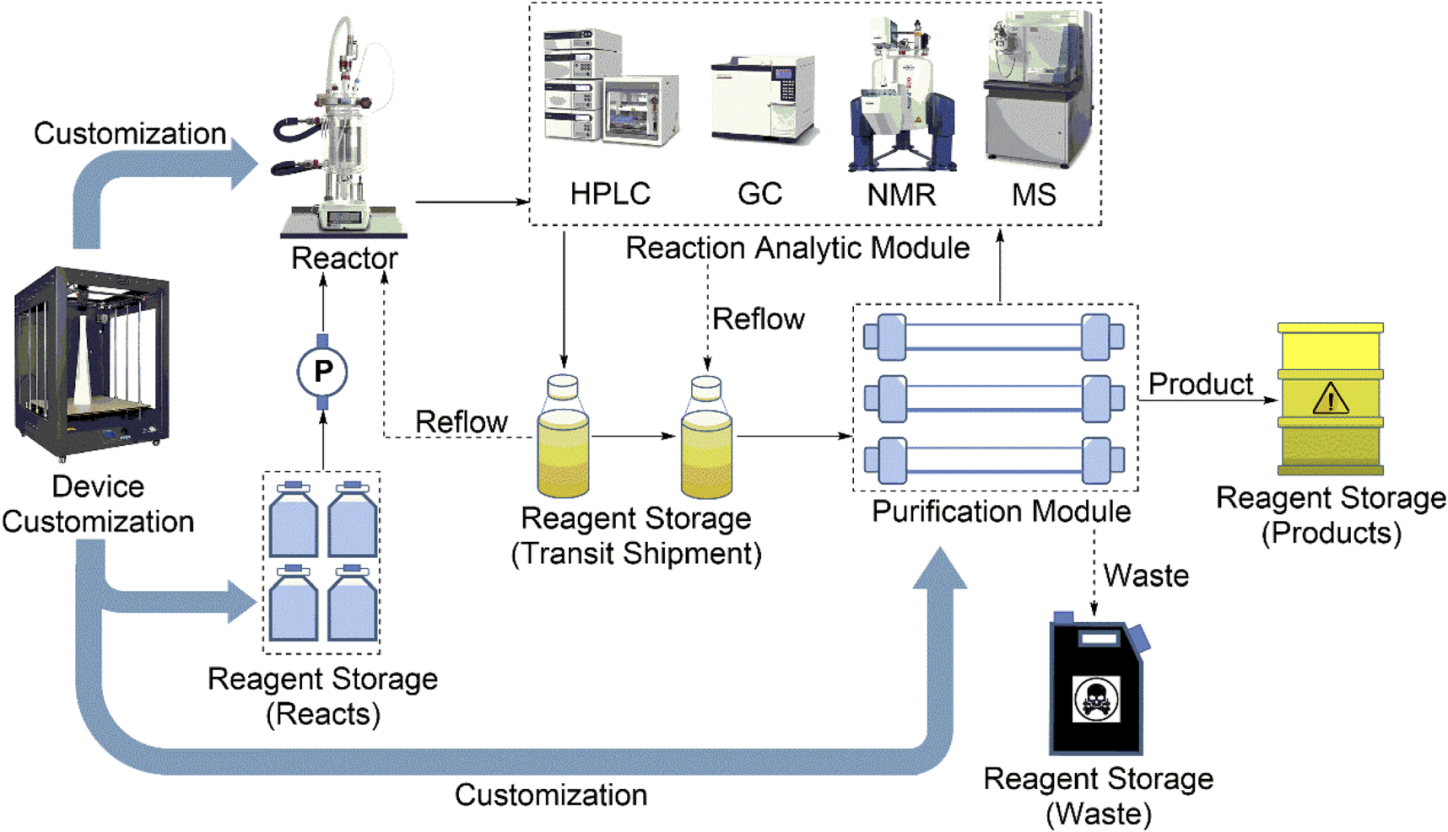
The configuration of the hardware includes five modules: reagent storage modules, reactors, the reaction analytic module, the purification module, and the device customization module.

Traditionally, chemical syntheses are completed by manual operation. Nothing is more common than this. However, people, as traditional executors, have some disadvantages: (1) they incur large amounts of time and manpower costs; (2) they make errors; and (3) they pose security risks. Therefore, the traditional chemical synthesis laboratory is encouraged to do something to promote change. AI-controlled synthesis can be much safer and much faster and could greatly reduce human errors and guarantee the repeatability of experiments. In our opinion, the configuration of automated systems as hardware should have five modules (Fig. 3): reagent storage modules, reactors, the reaction analytic module, the purification module, and the device customization module.

#### 2.1.1 Reagent storage modules: storage and sampling area of reagents

As the storage and sampling device of the hardware, reagent storage modules generally have four uses (Fig. 3). First, reagent storage modules store the reaction starting materials and cooperate with the injection system to complete the injection operation according to the parameters. Second, reagent storage modules can temporarily store intermediate products. Third, reagent storage modules ultimately samples the reaction products from the purification module. Last, reagent storage modules can store waste. Therefore, the reagent storage modules can be made from laboratory bottles, gas/liquid tanks, injectors, and even waste tanks (Table 2). On the other hand, for some reactions that need special storage conditions (*e.g.*, anhydrous and oxygen-free), reagent storage modules must be loaded with some attachments such as condition generator. Certainly, as shown in Fig. 3, chemists can also achieve specific reagent storage by using the device customization module.

**Table 2:** Overview of the current hardware

Modules	Compositions	Techniques/methods	Maturity	Flaws
Reagent storage	(1) Laboratory bottles	(1) Anhydrous	Poor adaptability to solid reagents	(1) Reagent transport pipe or pump blockage
	(2) Gas/liquid tanks	(2) Oxygen-free		(2) Solid reagents easily clump
	(3) Injectors			(3) Difficult for grinding and precise weighing of solid reagents
	(4) Waste tanks			
Reactor	(1) Reaction kettles	(1) Heater	Poor adaptability to different reagents	(1) Reagent transport pipe or pump blockage
	(2) Condition makers	(2) Dehumidifier		(2) Blockage hindering or stopping the reaction
	(3) Pumps and pipe	(3) Deaerator		(3) General reactors are not suited to specific reactions
		(4) Reflux unit		
Analytic module	(1) Spectrometers	(1) IR, Raman, UV-vis	Low performance for real-time tracking	(1) Application of real-time tracking is lacking
		(2) GC, HPLC		
		(3) MS		
		(4) NMR		
Purification module	(1) Chromatographic column	(1) Chromatographic	Automatic technique is not mature	(1) Reagent transport pipe or pump blockage
	(2) Filter			(2) Purification processes sometimes need manual operation
	(3) Fractionation tube			
Device customization module	(1) 3D printer	(1) Printing	Optional methods are limited	(1) Optional methods are lacking
		(2) Machining		(2) Application promotion remains at a low stage, almost just for customization of specific reactors

#### 2.1.2 Reactor(s): place of reaction and connector of multistep reactions

The reactor is the heart of the hardware and comprises the main structure of an AI-controlled synthesis executing system. Therefore, the reactor should have two functions: (1) as a place of a reaction executing chemical reactions and (2) as a connector connecting multistep reactions.

For the first function, the reactor, as the area of reaction, conducts synthesis reactions. At this time, the reactor is similar to a reagent storage module and provides a place where the starting materials react, and the reaction environment needs to be adjusted according to the requirements of the reactions. However, there are some points that differ between the reactor as a place of reaction and reagent storage. The reactor as a place of reaction can be deemed a kind of reagent storage module, but it always needs some attachments (*e.g.*, a heater, dehumidifier, deaerator, and reflux unit) to achieve different reaction conditions.

For the second function, the reactor is used as a connector connecting multistep reactions. When a synthesis reaction is completed in the reactor, the products should be moved into the next area (it can be the final reagent storage module or the next reactor). For example, Angelone *et al.* employed the same reactors and reused them multiple times to perform multistep synthesis reactions.^35^ At this time, the reactor acts as a connector. Therefore, the connector can also be deemed an important part of the reactor.

#### 2.1.3 Reaction analytic module: tracker of the reaction process

Regardless of manual operation in general laboratories or AI-controlled operation in automation laboratories, chemists always care about a key question: how is my reaction going? Therefore, it is necessary to use a reaction analysis module in the AI-controlled synthesis system. The reaction analysis module allows for the real-time monitoring of chemical process transitions by applying flow nuclear magnetic resonance (NMR),^28^ Fourier transform infrared (FT-IR) spectroscopy,^36^ UV-visible (UV-vis) spectrophotometry,^37^ electrospray mass spectrometry (ES-MS),^38^ liquid chromatograph-mass spectrometry (LC-MS),^39^ and high-performance liquid chromatography (HPLC)^40^ techniques. There are already advances being made in the development and application of reaction analytics modules. Cronin *et al.* reported real-time online NMR to create a self-optimizing reactor system for data analysis.^41,42^ Among them, LC-MS, NMR and HPLC are more compatible with automated synthesis platforms because of their good selectivity, high separation efficiency, wide application range, and high detection sensitivity.

#### 2.1.4 Purification module: purification and collection area of the main product

When a synthesis reaction or even a step of the synthesis reaction is completed, chemists need to purify the product(s) with which they want to obtain the final main product or perform the next reaction. Therefore, AI-controlled synthesis systems need to have one or several purification module(s). For example, Grob *et al.* used the stability of *N*-methyl iminodiacetic acid (MIDA) boronated under different reaction conditions, and different reagents and byproducts could be purified by the same process.^43^ The purification module generally uses chromatographic techniques to isolate or purify products. This means that the purification module usually works with the reaction analytics module. However, the purification module technique is not mature, and the purification process sometimes requires manual operations.^44^

#### 2.1.5 Device customization module: flexible designer of the hardware

Generally, normal devices are enough to support most chemical synthesis reactions. However, chemists still need to build some specific devices for carrying out some reactions under specific conditions. For example, Bubliauskas *et al.* used a 3D printing technique to build different specific reactors for 3 different chemical synthesis reactions and 11 chemical processes. This work has decreased negative effects from reactors, thereby avoiding frequent modification of chemical codes of the software for different reactions.^45^ Tailoring specific reactors for different chemical reactions provides a new way of choosing suitable reactors for the hardware. For a mature AI-controlled system, the device customization module should be stronger. In addition to customizing specific reactors, the device customization module can also design and build specific components for reagent storage modules and the purification module.

### 2.2 Applications and flaws of current hardware

The hardware of the AI-controlled synthesis system should be made from the five parts discussed above. There are some applications that have been published. Perera *et al.* reported an automated flow-based synthesis platform for nanomole-scale reaction screening and micromole-scale synthesis.^33^ Taking into account the potential susceptibility of the assessed chemical to air and moisture, the reactor was configured in a glovebox (<20 ppm O_2_, <20 ppm H_2_O). A convenient and efficient purification module could greatly reduce manual work. Rougeot *et al.* described a device that enabled automatic sampling of soluble components (solutions only) and slurries (solids and solutions) in parallel.^46^ Liu *et al.* developed an automatic platform “SPS-flow” for processing solid-phase synthesis. By employing the attachment high-pressure gas pump, SPS-flow completed the solid-phase synthesis reaction in six steps in 32 h and obtained a good yield (65%). This work opened a door for the subsequent development of AI-controlled solid-phase synthesis.^47^

However, existing systems are not perfect. There are some problems with the hardware (Table 2). Table 2 shows that the main flaw is the blockage of pipes or pumps with slurry. Flow system clogging includes several types: (1) crystallization of dissolved salts; (2) oil phase of the multiliquid phase; (3) particle deposits/bubbles present in the fluid; (4) chemical reaction (such as polymerization or decomposition) with wall materials or reactants, leading to solid precipitation. Therefore, precipitates formed during the reaction lead to irreversible blockage of reactor microchannels. This flaw has become a main barrier to the applications of AI-aided synthesis in general laboratories. Other flaws are mainly from devices with imperfect technologies. For example, some general reaction devices are not suited to specific reactions that need customized devices, the real-time tracking technique of the reaction analytics module needs to be improved, and manual operations of the purification module need to be resolved. Regardless of the flaw, they have reduced the performance of the hardware. Future hardware should deal better with different phases (especially solid), and require the seamless integration of sampling, reaction operations, purification separations, and analysis procedures.

## 3 Software: controller of AI-controlled synthesis

The software is the most important part of the AI-controlled synthesis system. As the center, the software not only stores and processes various data but also controls the behaviors of the hardware. This means that the AI-controlled synthesis system will be a lifeless system if there is no software. Therefore, the software should have the functions of recording, storing, processing and invoking data.

### 3.1 The configuration of the software

**Fig. 4:**
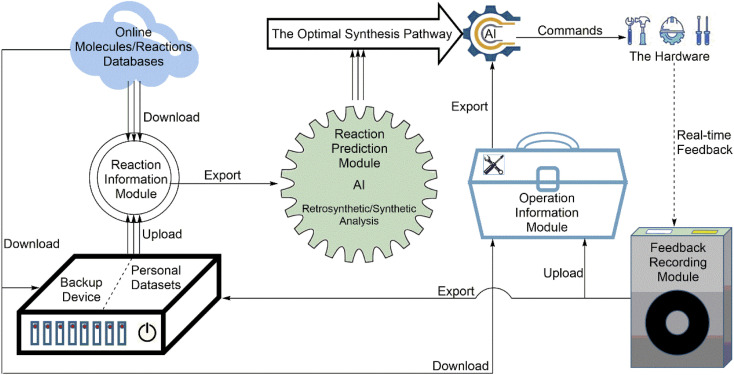
The configuration of the software includes four parts: the reaction information module, reaction prediction module, reaction operation information module, and feedback recording module.

The software is the core/brain of the system and the prerequisite for automation to become a reality. Generally, the software in automated systems for synthesis has three main functional categories: (1) controlling the hardware operating the synthesis reaction, (2) monitoring/analyzing the synthesis process, and (3) designing the synthesis strategy. To date, chemical synthesis, especially organic synthesis, is still a labor-intensive experimental subject. Robotic systems for the chemical domain are becoming increasingly popular due to the lack of general chemical programming languages and the ability to directly access natural language documents. A series of powerful AI algorithms are emerging quickly. They will perhaps make chemical synthesis no longer dependent on excessive human intervention. The rapid development of AI technology has brought rapid progress in experimental automation. The software should be made from four software modules (Fig. 4): (1) the reaction information module; (2) the reaction prediction module; (3) the reaction operation information module; and (4) the feedback recording module.

#### 3.1.1 Reaction information module: database of molecules and reactions

The reaction information module is the chemical reaction information exchange hub of the software. It obtains, stores, and invokes the data information of different molecules and reactions. There are many online molecule and reaction databases (*e.g.*, SciFinder, Reaxys, Chemical reactions from US patents (1976–Sep 2016) and so on). Therefore, the reaction information module simply needs to connect to the internet and download data from online databases.^48–52^ Sometimes, chemists can also build personal reaction datasets and upload them to the reaction information module. For example, Coley *et al.* used deep learning with a Transformer Encoder module to get reaction and operation information directly from the published papers,^53^ and then developed the open-access program Open Reaction Database (ORD) as a general database for getting reaction information, so that chemists can get chemical information more easily.^54^ These studies developed a new way by which the software can also get reaction information from literature studies, not only from databases.

Moreover, the reaction information module should have a backup device as an attachment. When a network failure happens, the backup device can upload data to the reaction information module. If the main body of the reaction information module does not work, the backup device can also get data directly from online databases.

#### 3.1.2 Reaction prediction module: providing the optimal synthesis pathway

This module is the core of the software. The reaction prediction module, as a storing and invoking module, collects information on different reaction mechanisms. Simultaneously, the reaction prediction module must be used as a data processing module to obtain the export data from the reaction information module and to find an optimal synthesis pathway for the hardware. This means that the reaction prediction module needs to integrate an AI program to predict the synthesis pathway. The reaction prediction module mainly has two types: (1) retrosynthetic analysis and (2) forward-synthetic analysis.

Retrosynthetic analysis is more frequently used by current programs^46^ because chemists often know products more than reactants. Many retrosynthetic analysis programs based on AI are available. For example, Automated System for Knowledge-based Continuous Organic Synthesis (ASKCOS) is a frequently used AI retrosynthesis prediction program.^55^ Other programs such as IBM RXN,^56^ Chemical.AI,^57^ and Molecule.one^58^ are famous programs. IBM RXN is also the first free AI web service for predicting chemical reactions. Forward-synthetic analysis is actually a reaction mechanism inference and is commonly used in *ab initio* or quantum-based prediction.^59,60^ AI-based forward-synthetic prediction is less common than retrosynthetic analysis. Although forward-synthetic analysis programs are few, it does not mean that there is no program to perform forward-synthetic analysis in the AI field. ASKCOS can also perform forward-synthetic analysis. New reaction prediction programs have also been developed recently. For example, researchers from AstraZeneca developed two AI-based open-source prediction programs REINVENT^61,62^ and GraphINVENT^63–65^ for guiding new drug molecule synthesis and exploring new chemical places of new drug molecules.

#### 3.1.3 Reaction operation information module: processing hub of operation data

The data of the reaction operations are the same as those of the manual experimental workflow. These include two aspects: (1) the conditions and environments of a chemical synthesis reaction (*e.g.*, temperature, pressure, pH, catalyst, and solvent) and (2) the operations of a chemical synthesis reaction (*e.g.*, dosage, adding order of reactions, and order of operations). Therefore, the operation information module can obtain these data in three ways: (1) directly from online databases, (2) from the reaction information module, and (3) from the feedback information module to obtain the real-time feedback of the hardware (Fig. 4). Fig. 4 also shows that the export data from the operation information module mix with the export data from the reaction prediction module and become a command to control the hardware. Therefore, the reaction operation information module is the toolbox of the software.

#### 3.1.4 Feedback recording module: processing real-time data from the hardware

When the hardware is working, it can generate log files as feedback data to send back to the software. These feedback data are similar to the experimental data in general laboratories, which are very important and need to be recorded and processed. Hence, the software needs the feedback information module to record the feedback data from the hardware. As shown in Fig. 4, the feedback recoding module has three main functions: (1) obtaining the real-time feedback data from the hardware; (2) uploading the real-time operation data to the reaction operation information module; and (3) invoking the real-time experimental data into the personal dataset to improve the reaction information module.

The feedback recording module first acts as a scout base for the software. It can be based on real-time feedback data from the hardware and help the software modify old commands or send new commands to the hardware. Therefore, the software can also use the feedback data from the hardware to improve its performance. Therefore, the feedback recording module plays a key role in supervising machine learning and improving software strategies.

### 3.2 Applications and flaws of current software

Recently, a series of powerful functions of AI algorithms have been rapidly developed in parallel, and they could greatly facilitate the development of platforms that could automate custom general synthesis routes. There are many applications for the software to be reported. For example, Burger *et al.* developed an AI robot named the mobile robotic chemist and proposed the concept “AI Chemist”. AI Chemist can complete research on approximately 1000 precatalyst compounds a week, which is a burdensome task that a PhD student needs 4 years to complete.^66^ Cronin *et al.* developed a general system for chemical digitization and automatic synthesis that could automatically read the literature.^67^ This is another epoch-making achievement in the field of AI-based chemistry following the Chemputer.^68^ By combining Chemical Description Language (χDL) and the easy-to-use interface ChemIDE, ChemIDE allows users to program chemical synthesis without any coding knowledge. This “paper in, product out” AI chemist took synthesis automation to a new level. Surprisingly, Stuyver *et al.* reported this year that they combined QM with GNN learning to develop an ml-QM-GNN module for forward-synthetic analysis. The ml-QM-GNN module can not only predict the activation energy, reactivity, and chemoselectivity of reactants but also find the preferred reaction mode.^69^ This work provided a new way of developing forward-synthetic analysis methods with a combination of *ab initio* and machine learning. Applications of software are becoming increasingly widespread. Software is the brain of automated platforms that help chemists control the entire synthesis process. Existing software has much room for improvement in designing reaction routes.

There are still some flaws in the software that need to be considered. In Table 3, we find that the current software as a controller of AI-controlled synthesis systems has two main flaws: (1) AI-based forward-synthetic analysis programs are few; (2) weakly integrating computer-aided synthesis planning (CASP) tools; and (3) the data of failed syntheses are not attended to. Many chemists want to know the synthesis route for a product. Retrosynthetic analysis has been developed and used widely. However, many laboratories focus on developing new synthesis methods and generating new compounds. Traditional forward-synthetic analysis is mainly based on QM calculations but not AI calculations. This hinders AI-controlled synthesis systems in laboratories that are interested in finding new synthesis methods and new compounds. Because pure retrosynthetic analysis can obtain many synthesis pathways with many different reactions, many of the predicted synthesis pathways are unhelpful for some general chemosynthesis laboratories. Therefore, a mature forward-synthetic analysis with special reactants can guide these general chemosynthesis laboratories to generate new compounds or to explore new synthesis methods. This is more helpful than retrosynthetic analysis to increase the success rate of synthesis experiments in wet laboratories. Some software weakly integrates CASP tools. This makes the software rely too much on the internet (*e.g.*, online CASP tools and online databases). Moreover, the data of failed syntheses as feedback can make the software avoid previous mistakes. Thus, software with more kinds of synthetic analysis, including CASP tools, should receive more attention in the future.

**Table 3:** Overview of the current software

Modules	Data source	Main jobs	Maturity	Flaws
Reaction information module/operation information module	(1) Online databases	(1) Obtaining and processing data	The information modules are overly dependent on online databases, but the backup device is not valued	(1) Many databases are not open source
	(2) Personal datasets	(2) Storing data		(2) The software integrates online databases weakly
		(3) Invoking data		(3) The backup device is not valued
				(4) User-friendliness is insufficient
Reaction prediction module	(1) Machine learning	(1) Synthetic analysis	Retrosynthetic analysis is used widely, but AI-based forward-synthetic analysis is lacking	(1) Few AI-based forward-synthetic analysis programs are used
	(2) QM calculations	(2) Finding an optical synthesis path out		(2) The software integrates CASP tools weakly
	(3) *Ab initio* calculations			(3) User-friendliness is insufficient
Feedback recording module	(1) The hardware	(1) Obtaining real-time feedback data from the hardware	The data of failed syntheses are usually discarded	(1) The data of failed syntheses are not valued
		(2) Sending data to information modules		

## 4 Automation control component: the bridge between software and hardware

We have pointed out that the software controls every behavior of the hardware to perform AI-controlled synthesis. Chemists need a/some device(s) to connect the software and the hardware (Fig. 2). For a control system, the automation control component (also called the digitization control component) is usually the first choice. There are some advantages to the automation control component: (1) mature technology; (2) various implementation methods; and (3) running stably. The automation control component is generally embedded. This means that the automation control component does not need to occupy a larger space. Currently, people can directly use microcontroller units or employ integrated chips or even integrated programs into the software.

**Fig. 5:**
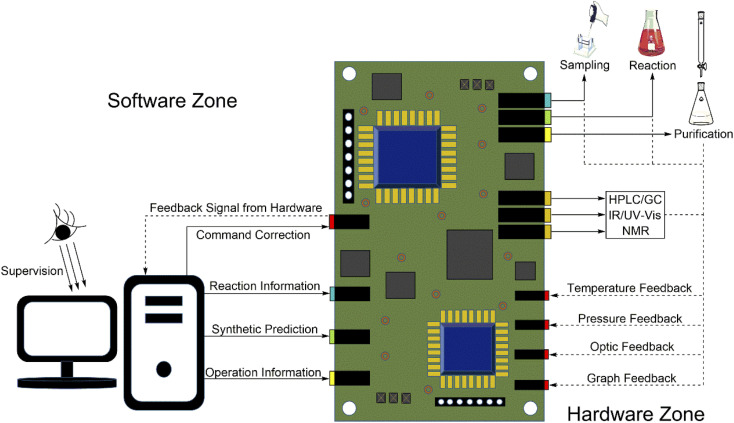
The automation control component is the bridge between the software and the hardware.

As shown in Fig. 5, we can directly know the working principle of the automation control component. First, we introduced that the software controls the hardware to execute synthesis reactions in the AI-controlled synthesis system. As shown in Fig. 5, the software sends commands that include reaction and operation information and synthetic prediction information to the automation control component. Then, the automation control component processes them and hands them to the hardware to perform sampling, reaction, purification, *etc.* Second, we note that the software should have a feedback information module to obtain feedback information from the hardware. Actually, the automation control component also plays the role of a sensor. The different original feedback signals mainly come from the hardware and are input to different sensor units of the automation control component. Then, the automation control component processes these feedback signals and sends them to the feedback information module of the software so that the software can more flexibly correct the controlling strategies. Overall, the automation control component is a connected hub that connects the software and the hardware.

After introducing the working principle of the automation control component, we obtain a question. Which type of automation control component has been used for connecting the software and the hardware? Hammer *et al.* reported that chemists mainly use microcontroller units with Python or C++ codes to control the hardware for simple synthesis.^70^ Prabhu *et al.* pointed out that chemical researchers can choose multiple types of microcontroller units to make the software control the hardware (Fig. 5). Chemists can directly use microcontroller boards (*e.g.*, Arduino 101, Ardbox PLC, chipKIT Uno32, and Particle Argon) or choose single-board computers (*e.g.*, Raspberry Pi 3 and BeagleBone Black).^71^ Wilbraham *et al.* reported that they utilized χDL to drive the microcontroller unit “chemical processing unit (ChemPU)” for controlling, optimizing, and exploring synthesis reactions.^72^ In this year, Cronin *et al.* improved the compatibility between χDL and ChemPU. They employed this more mature automatic platform to process 53 kinds of chemical reactions, which are among the 103 kinds of reactions that are encoded by χDL, and obtained good results and high yields. This work makes it possible to replicate chemical synthesis experiments from previous literature, and also greatly improves the performance of current AI-controlled synthesis systems.^73^ The real jobs of chemists are just to rewrite or to modify codes in C++, Python, or even in their own machine languages.

According to these reports above, we find that an automation control system can already be executed on a personal computer. But there are still two unfavorable factors around the automation control system in current AI-aided synthesis platforms: (1) the technical barrier is high for general chemists; (2) open-source application programming interfaces (APIs) of commercial programs on third-party platforms are lacking. Although applications of the automation control system may have been sufficient for the automation control component of AI-aided synthesis, the development of a more intelligentized automation control component of AI-aided synthesis should become chemists' focus in the future.

## 5 Perspective and outlook

From the contents above, we can find that AI-aided synthesis systems are increasingly widely used in the chemical synthesis field. However, the rate-determining step of AI-controlled synthesis systems is rarely mentioned, which makes it difficult to apply in general laboratories. Recently, many chemists have been developing new devices for AI-controlled synthesis and trying to improve the performance of AI-controlled synthesis systems. Therefore, we have some perspectives for the subsequent development of AI-controlled synthesis systems.

We propose some viewpoints for the hardware of AI-controlled synthesis systems:

(1) More adaptable. The hardware should execute the synthesis reaction in different phases. It needs to add a new main module as a cleaner to clean blockage from solid or multiliquid reagents. The module can be an ultrasonic generator or a high-pressure air/liquid pump.

(2) More customization devices. General devices are sometimes not suited to some specific synthesis reactions. This is a hindrance to AI-controlled synthesis in general laboratories. In addition to 3D printing techniques, more methods of updating or even building specific devices should be developed.

(3) More automatic. Manual operations of the purification module reduce the automatic performance of the hardware. The purification module should load flow controllers and automatic replacement devices to simulate and replace chemists' hands.

(4) Quicker. Real-time tracking is important for the process of a reaction. The reaction analytic module should be quicker to analyze the situation of products.

(5) More convenient. An automated chemical operation platform should save the time cost of chemical experts. The system should not be too complicated such that it would require much training for operators to operate the equipment.

(6) Lower economic investment. Fund-rich laboratories are not everywhere. Thus, lower cost is helpful to make AI-controlled synthesis migrate into general laboratories.

We propose some viewpoints for the software of AI-controlled synthesis systems:

(1) AI-based forward-synthetic analysis. The development of forward-synthetic analysis programs should be attended to. Many general laboratories focus on exploring new synthesis methods and new products from specific reactants. Predicting possible products from known reactants is necessary. On the other hand, the success rate of choosing the right synthesis reaction path is higher with forward-synthetic analysis. Hence, they need forward-synthetic analysis rather than retrosynthetic analysis. Forward-synthetic analysis has some advantages that retrosynthesis analysis does not have. It is helpful to extend AI-controlled synthesis systems into laboratories that focus on developing new synthesis methods or discovering new compounds.

(2) Paying attention to negative data. Chemists generally pay attention to the positive data that they obtain, but the negative data (*e.g.*, infeasible reaction data and low yield reaction data) are always skipped. Therefore, the reaction information module of the software should add the information on negative data. Negative data can offer failed examples for the AI-controlled synthesis system so that the AI-controlled synthesis system can avoid unnecessary mistakes.

(3) Combined with CASP tools. At present, chemical automation studies focusing on synthesis planning are rarely reported, and they are much more complicated than the automation of the synthesis process. Thus, an automated system that includes CASP tools should receive more attention in the future.

(4) Greater interactivity. The software should be able to intervene in the entire reaction process in a timely manner while monitoring the reaction process to ensure the synthesis effect and avoid potential safety hazards.

(5) User-friendliness of the CASP programs integrated into automated systems. The existing command line programs may be suitable for chemo-informatics, but some chemists use graphical user interfaces (GUIs).

## 6 Conclusion

A series of powerful advancements in the hardware and software of AI-controlled synthesis systems are emerging quickly and in parallel. They have the potential to make synthesizer development possible and automate custom and general synthesis routes. AI-controlled synthesis has the following advantages: (1) safety; (2) high speed; (3) reproducibility; and (4) low cost of synthesizing compounds. We have pointed out some limiting factors that mean AI-controlled synthesis systems cannot be extended to general laboratories. The hardware of the AI-controlled synthesis system should include a cleaner module to keep the hardware channels open. Regardless of solid or multiliquid reagents, the improved hardware can use the cleaning module to clean blockages in channels. For the software of the AI-controlled synthesis system, its reaction prediction module should have more mature AI-based forward-synthetic analysis functions. It is helpful to extend the AI-controlled synthesis system into general laboratories that are interested in developing new organic synthesis methods. In summary, an AI-controlled synthesis system could quickly and accurately control reaction conditions such as temperature and pressure to handle sensitive chemicals and dangerous reactions more safely.^74–76^ Fortunately, some groups are trying to overcome these challenges from the flaws of current AI-controlled synthesis systems and have made some achievements.^35,47,69,73^ Beyond all doubt, with the development of AI algorithms of the software and engineering technology of the hardware, we believe that chemists will quickly move toward the goal of AI-controlled synthesis. Therefore, extending the method of AI-controlled synthesis into general laboratories should not take much longer.

## Funding

This work was supported by the National Natural Science Foundation of China (32125033) and the Program of Introducing Talents of Discipline to Universities of China (111 Program, D20023).

## Author contributions

Mr Wei Wang wrote mainly the manuscript. Mr Yingwei Liu and Prof Gefei Hao provided guidance on knowledge of machine learning algorithms. Mr Wei Wang, Mr Zheng Wang and Prof. Baoan Song provided support on knowledge of chemical synthesis. Prof. Gefei Hao and Prof. Baoan Song provided suggestions to improve and modify the manuscript.

## Conflicts of interest

There are no conflicts to declare.

## Supplementary Material
